# Genomic Analysis Reveals the Molecular Basis for Capsule Loss in the Group B *Streptococcus* Population

**DOI:** 10.1371/journal.pone.0125985

**Published:** 2015-05-06

**Authors:** Roberto Rosini, Edmondo Campisi, Matteo De Chiara, Hervé Tettelin, Daniela Rinaudo, Chiara Toniolo, Matteo Metruccio, Silvia Guidotti, Uffe B. Skov Sørensen, Mogens Kilian, DEVANI Consortium, Mario Ramirez, Robert Janulczyk, Claudio Donati, Guido Grandi, Immaculada Margarit

**Affiliations:** 1 Novartis Vaccines and Diagnostics Srl, Siena, Italy; 2 Institute for Genome Sciences, University of Maryland School of Medicine, Baltimore, Maryland, United States of America; 3 Department of Biomedicine, Aarhus University, Aarhus, Denmark; 4 Instituto de Microbiologia, Instituto de Medicina Molecular, Faculdade de Medicina, Universidade de Lisboa, Lisbon, Portugal; 5 Department of Computational Biology, Research and Innovation Centre, Fondazione Edmund Mach, Via E. Mach 1, San Michele all'Adige, Italy; Instituto Butantan, BRAZIL

## Abstract

The human and bovine bacterial pathogen *Streptococcus agalactiae* (Group B Streptococcus, GBS) expresses a thick polysaccharide capsule that constitutes a major virulence factor and vaccine target. GBS can be classified into ten distinct serotypes differing in the chemical composition of their capsular polysaccharide. However, non-typeable strains that do not react with anti-capsular sera are frequently isolated from colonized and infected humans and cattle. To gain a comprehensive insight into the molecular basis for the loss of capsule expression in GBS, a collection of well-characterized non-typeable strains was investigated by genome sequencing. Genome based phylogenetic analysis extended to a wide population of sequenced strains confirmed the recently observed high clonality among GBS lineages mainly containing human strains, and revealed a much higher degree of diversity in the bovine population. Remarkably, non-typeable strains were equally distributed in all lineages. A number of distinct mutations in the *cps* operon were identified that were apparently responsible for inactivation of capsule synthesis. The most frequent genetic alterations were point mutations leading to stop codons in the *cps* genes, and the main target was found to be *cpsE* encoding the portal glycosyl trasferase of capsule biosynthesis. Complementation of strains carrying missense mutations in *cpsE* with a wild-type gene restored capsule expression allowing the identification of amino acid residues essential for enzyme activity.

## Introduction

*Streptococcus agalactiae* (Group B Streptococcus, GBS) colonizes the gastrointestinal and/or genital tract of about 20% of women and can cause neonatal sepsis and meningitis with high mortality rates. It is also an important cause of morbidity in the elderly and immunocompromised adults, and of bovine mastitis [1]. The polysaccharide capsule of GBS is an important virulence factor conferring anti-phagocytic properties. Low anti-capsular antibody titers correlate with increased risk of disease, and capsular polysaccharide-based vaccines are currently under development [2]. The synthesis of the GBS capsular polysaccharide (CPS) requires multiple enzymes encoded in the *cps* operon. This gene cluster shares homologies with operons from other encapsulated Gram-positive bacteria [3] and with those responsible for the synthesis of the lipopolysaccharide O-antigen in Gram-negatives [4]. In all these bacteria, the polysaccharide repeating unit is synthesized on the membrane acceptor undecaprenyl phosphate by multiple glycosyl transferases, transported outside the cell membrane by a translocase and assembled into high molecular weight polymers [3].

GBS can be classified into 10 distinct serotypes varying in polysaccharide composition, based on reactivity with specific antisera by using Lancefield precipitation or latex agglutination tests [5]. Yet, some isolates are considered as non-typeable (NT) because they do not react with any anti-capsular serum. The frequencies of detected NTs in GBS epidemiological studies have recently decreased with the development of serotyping methods of improved sensitivity [6,7]. However, they still account for 5–20% of the isolates colonizing or infecting human adults [8] and for 30–77% of those obtained from bovine mastitis [6,9–11].

The GBS NT phenotype could in principle be due to low or complete lack of capsule expression, or to the presence of yet unknown polysaccharide variants that do not react with the available typing antisera [6]. The molecular basis for the absence of capsule expression, and the genetic relationship between NT and encapsulated GBS strains have been poorly investigated to date. Newly developed molecular typing methods based on PCR amplification of the different GBS *cps* alleles have allowed assigning capsular genotypes to most NT strains [6,12,13]. Ramaswamy *et al*., [14] identified an IS1381 insertion in the *cpsE* gene as possibly responsible for the NT phenotype of a capsular genotype V strain. Furthermore, a single strain lacking the entire capsule locus was recently described [15].

Here we undertook a comprehensive study of the GBS non-encapsulated population by genomic analysis of a large collection of colonizing and invasive strains obtained both from humans and cattle. To achieve a high-resolution characterization of the GBS population structure and to map the distribution of NT isolates among the different lineages, we constructed phylogenetic trees based on genome Single Nucleotide Polymorphisms (SNPs) or Multiple Locus Sequence Typing (MLST), including in the analysis all publicly available sequenced strains. Furthermore, the different types of genetic alterations leading to capsule loss and the main target genes of these mutations were identified by DNA sequence comparison with encapsulated reference strains.

## Materials and Methods

### Ethics Statement

All animal studies were carried out in compliance with current Italian legislation on the care and use of animals in experimentation (Legislative Decree 116/92) and with the Novartis Animal Welfare Policy and Standards. Protocols were approved by the internal "Novartis Animal Ethical Committee" (approval number: AEC 200825) and authorized by the "Italian Ministry of Health" (authorization number: 21/2009-B).

### Bacterial strains and culture conditions

A total of 206 strains that were non-typeable by latex agglutination methods (Group B kit, no 73259, SSI diagnostic, Copenhagen, Denmark; GBS serotyping kit, Essum, Umeå, Sweden) were included in the study. Fifty strains from healthy pregnant women were collected, identified and serotyped during the European DEVANI study (2009–2010, Italy, Germany, Spain, Denmark, Belgium, Bulgaria, Czech Republic, UK) [16]. Thirty-six strains were obtained in Portugal from adult carriers or from patients with invasive GBS disease [17]. Ten strains were obtained from bovine mastitis in Denmark. For 96 of these strains the lack of capsule was confirmed by flow-cytometry, and they were subjected to deep genome sequencing. The final dataset of sequenced strains also included 32 encapsulated reference strains of serotypes Ia (n = 7), Ib (n = 5), II (n = 6), III (n = 10), V (n = 4) from both colonized and infected neonates (S2 Table). Bacteria were grown at 37°C in Todd Hewitt broth (THB, Difco Laboratories), or in trypticase soy agar supplemented with 5% sheep blood.

### Isolation of genomic DNA

For genomic DNA isolation and purification, bacteria were grown overnight at 37°C in THB. Chromosomal DNA was prepared from 2 mL of culture using the GenElute Bacterial Genomic DNA Kit (Sigma) according to the manufacturer’s instructions. DNA concentration was estimated by optical density determination at 260nm.

### Deep sequencing of genomic DNA

Whole genome sequencing was performed using either of the two methods described below.

*Method A*: 5 μg of bacterial DNA randomly sheared by means of Covaris S2 focused ultrasonicator into fragments of 300 bp on average. Genomic libraries were automatically generated starting from 2.5 μg of sheared DNA by means of SPRIworks Fragment Library Kit I cartridges (Beckman Coulter) and barcoded with TruSeq indexes (Illumina), with a 300–600 bp size selection. Library enrichment was performed using a TruSeq Sample Preparation Kit (Illumina) and amplified products were purified by Ampure XP Magnetic Beads (Beckman Coulter) according to manufacturer’s protocol. Adapted libraries were quantified by qPCR using the “KAPA SYBR FAST (ABI Prism) Kit” (KAPA) and the 7900HT Fast RT-PCR System (Life Technologies), to allow optimal cluster density of reads and equimolar multiplexing of libraries. Libraries were then pooled, denatured and diluted to a final concentration of 6 pM, and loaded on the cBot clonal amplification system (Illumina) for cluster generation. A deep sequencing run was later performed on a HiSeq2500 platform using TruSeq SBS chemistry (Illumina) to generate 2x100 bp paired-end reads.

*Method B*: DNA libraries were constructed for sequencing on the Illumina platform using the NEBNext DNA Sample Prep Master Mix Set 1 (New England Biolabs, Ipswich, MA). First, DNA was fragmented with the Covaris E210. Then libraries were prepared using a modified version of manufacturer’s protocol. The DNA was purified between enzymatic reactions and the size selection of the library was performed with AMPure XT beads (Beckman Coulter Genomics, Danvers, MA). The PCR amplification step was performed with primers containing an index sequence seven nt in length.

Adapted libraries were quantified by qPCR using the “KAPA Library Quantification Kit” (KAPA) and the ViiA 7 RT-PCR System (Life Technologies), to allow optimal cluster density of reads and equimolar multiplexing of libraries. Libraries were then pooled, denatured and diluted to a final concentration of 10.5 pM, and loaded on the cBot clonal amplificationsystem (Illumina) for cluster generation. A deep sequencing run was later performed on a HiSeq2000 platform using TruSeq SBS chemistry (Illumina) to generate 2x100 bp paired-end reads.

### Flow cytometry analysis

Flow cytometry (FACS) experiments using anti-capsular (Ia, Ib, II, III and V) monoclonal antibodies were conducted as described elsewhere [18] with minor differences. Briefly, bacteria grown in THB to exponential phase were harvested and fixed in PBS containing 0.1% (w/v) paraformaldehyde (PFA). The fixed cells were washed with PBS and incubated for 1 h at room temperature with secondary antibody alone or immune mouse monoclonal antibodies raised against purified polysaccharides, diluted 1:200 in PBS containing 0.1% BSA. The cells were incubated for 1 h at room temperature with R-phycoerythrin-conjugated F(ab)2 goat anti-mouse immunoglobulin G, diluted 1:100 in PBS containing 0.1% BSA. All data were collected using a BD FACS CANTO II (BD Bioscience) by acquiring 10,000 events, and data were analyzed using the Flow-Jo software (v.8.6, TreeStar Inc.).

### Genome Assembly and Annotation

The draft genome sequences of 96 NT isolates and 32 typeable isolates of GBS (S2 Table) were assembled using Celera Assembler 7.0 [19]. Each genome was assembled using raw read coverage ranging from 40 to 150x. Criteria for selection of the best assembly were the highest coverage possible resulting in the lowest number of contigs/scaffolds with a genome size (cumulative size of contigs) closest to the average GBS genome size. The draft genomes were annotated using the Institute for Genome Sciences CloVR-Microbe pipeline [20]. The newly sequenced genomes were deposited in the NCBI database under the Bioproject ID PRJNA278931.

### Genome alignments and SNPs selection

All the sequenced genomes were aligned to the reference complete genome of the strain 2603V/R using the program Nucmer from the Mummer suite [21]. From these, the SNPs in the pairwise alignment were identified using the command “show-snps” (parameters –ClrHI) of the MUMmer software suite. Using custom scripts, the list of polymorphic sites was then filtered to include only sites in the core genome of GBS, *i*.*e*. those regions of the sequence of the reference strain 2603V/R that could be aligned against all other strains, including 0.42Mbp. The alleles of all strains in the polymorphic sites were determined using pair-wise sequence alignments against the reference 2603V/R strain. Using this procedure, we identified 14,092 SNPs.

### Generation of a MST tree

Sequence Types (STs) of our GBS strain collection were assigned on the basis of FASTA analysis against the 7 reference housekeeping genes present in the GBS MLST database (http://pubmlst.org/sagalactiae/) (S2 Table). A minimum spanning tree (MST) based on the goeBURST algorithm was generated by integrating these data with the ST-types present in the GBS MLST database using the PHYLOVIZ program [22].

### Sequence alignments and phylogenetic analysis

The *cps* loci were extracted from sequenced genomes and analyzed by sequence alignment using MUSCLE algorithm implemented on Geneious suite (Geneious R7, created by Biomatters, available from http://www.geneious.com/). The BLAST algorithm [23] was used to assign capsular type using reference *cps* loci. To search for mutations events the *cps* locus from each of the sequenced NT isolates was aligned to that extracted from its serotype-specific encapsulated reference strain genome (Serotype Ia (A909, NC_007432), Ib (H36B, NZ_AAJS00000000.1), II (ES-PW-160, this study), III (COH1, HG939456) and V (2603 V/R, NC_004116).

Phylogenetic and evolutionary analysis was performed by the Neighbor-Joining method using MEGA6 [24] with 100 bootstrap replicates. The evolutionary distances were computed using the Maximum Composite Likelihood method [25]. All positions containing gaps and missing data were eliminated.

For analysis of the GBS pan-genome, the coding sequences of internally sequenced and fully annotated genomes were clustered by the CD-HIT algorithm (http://weizhongli-lab.org/cd-hit/) using a 70% cut-off for protein identity over 70% of the length.

### Construction of plasmid pAM-*cpsE* vector and complementation analysis

The complementation vector pAM-*cpsE* was constructed using the primers *cpsE*-f CCTGTCATGCGGCCGCGAAAAAGGAAGTAAGGGGCTCTTGTATTG and *cpsE*-r CTCTCTCTGAGATCTCATTATATTCCTTTCAAACCTTACCTTTAC from GBS 515 Ia strain genomic DNA to amplify a 1,442 bp fragment. The PCR product was digested with NotI/BglII and ligated in pAM80 plasmid under the promoter of the GBS pilus 1 operon [26]. The resulting pAM-*cpsE* plasmid was transformed into GBS NT strains DK-PW-097, 325662, ES-PW-195, IT-PW-0094 and BE-PW-101. Transformations were performed essentially as described [27]. DNA plasmid concentration was optimized for each strain: ranging between 1 μg and 3 μg. Transformants were selected on TSA plates with 10 μg/ml of chloramphenicol at 37°C.

### RNA extraction and qRT-PCR

Bacteria were grown in triplicate in 10 ml THB at 37°C and harvested at OD_600_ = 0.4 (log phase). To rapidly arrest transcription, the cultures were cooled on ice and added to 10 ml of frozen THB medium in a 50 ml conical tube. Bacteria were collected by centrifugation for 15 min at 4000 rpm at 4°C, washed in 400 μl PBS added with 800 μl RNAprotect (Qiagen) and finally incubated in 100 μl TE buffer with 30 μg/ml lysozyme (Sigma) and 200 units mutanolysin (Sigma), for 40 min at 37°C with gentle agitation.

RNA was extracted using the Qiagen RNeasy Mini Kit (Qiagen) according to the manufacturer’s instructions. RNA samples were treated with DNase (Roche) for 2 h at 37°C and further purified using the RNeasy Mini Kit (Qiagen), including a second DNase treatment on the column for 30 min at room temperature, according to the manufacturer’s instructions. The cDNA was prepared by the Reverse Transcription System (Promega) by using 500 ng of RNA per reaction. Real time quantitative PCR (qRT-PCR) was performed on 50 ng of cDNA that was amplified using LightCycler 480 DNA SYBR Green I Master (Roche). Reactions were monitored using a LightCycler 480 instrument and software (Roche). For each biological replicate, three technical replicates were performed. To quantify the *cps* operon transcription levels, we used primers annealing on *cpsA* (cpsA-F/R and cpsAup-F/R) and *cpsE* (cpsE-F/R,). Primer sequences are reported in S1 Table. The transcript amounts were standardized to the housekeeping gene (*gyrA*) and compared with standardized expression in the wild-type strain (ΔΔC_T_ method).

## Results

### Identification of GBS non-encapsulated isolates for molecular analysis

The first step in the investigation of the molecular basis for the loss of capsule expression in *Streptococcus agalactiae* was the selection of a wide panel of strains devoid of capsule. GBS serotyping by Lancefield immuno-precipitation or by latex agglutination can indeed provide discrepant results regarding the NT phenotype [28,29]. To investigate capsule expression by a more accurate flow-cytometry based approach, we generated monoclonal antibodies against purified CRM-conjugated polysaccharides of the five most frequent capsular genotypes, *i*.*e*. Ia, Ib, II, III, V, [8,30]. In a control experiment, the obtained monoclonals specifically stained reference encapsulated strains of the five corresponding serotypes (S1A Fig); by contrast, none of the antibodies reacted with non-encapsulated strains of the same capsular genotypes (S1B Fig).

The developed flow-cytometry-based assay was then used to re-examine a collection of European strains previously reported as NT according to conventional serological methods. These strains were selected among those NT belonging to genotypes Ia, Ib, II, III, V, as determined by PCR analysis [13].

Among the 206 re-examined strains, 96 showed completely negative fluorescence signals and were chosen for the intended molecular analysis. These included 10 isolates from bovine mastitis, 59 from human adult carriers and 27 from adult invasive disease (S2 Table). The 110 remaining strains showed comparable levels of fluorescence to reference encapsulated bacteria, confirming a higher sensitivity of the used flow-cytometry assay compared to classical serotyping methods [28,29].

The relative distribution of confirmed non-encapsulated isolates among the five selected capsular genotypes reflected the relative prevalence of the corresponding serotypes in GBS colonizing and invasive strains from European epidemiological studies, with Ia, III and V being the most frequent genotypes, followed by II and Ib (Table 1). Concerning the ten examined NT strains obtained from cattle, 6 belonged to capsular genotype II while the other four genotypes were represented by a single isolate (S2 Table).

**Table 1:** Relative frequency of capsular genotypes Ia, Ib, II, III and V among our collection of NT isolates and in encapsulated GBS strains obtained from adults in Europe.

Study period	Origin	Typing methods	no. isolates	Prevalence by genocapsular types (%)					References
				Ia	Ib	II	III	V	
2002–2010	Invasive Disease	PCR	27	22	0	15	26	37	This Study
1996–2012	Invasive Disease	PCR\Serology	967	25	14	10	26	26	[17,31,32]
2002–2010	Carriers	PCR	59	17	8	10	34	31	This Study
1993–2006	Carriers	PCR\Serology	2363	22	13	16	32	18	[8]

### Phylogenetic structure of the GBS population and evidence of random distribution of human NT isolates among the different lineages

To undertake a population-scale molecular analysis of non-encapsulated GBS, the 96 selected NT strains along with 32 reference encapsulated isolates belonging to serotypes Ia (n = 7), Ib (n = 5), II (n = 6), III (n = 10) and V (n = 4) were subjected to whole genome next generation sequencing. *De novo* assembly produced DNA sequences ranging from 2.09 to 2.38 Mb and automatic annotation yielded 1940 to 2625 ORFs per genome.

SNP-based comparative analysis was conducted on the 128 obtained genome sequences from NT and encapsulated strains, plus 245 genomes downloaded from the NCBI database. The analysis was based on the alignment of 0.42 Mbp core sequences to the reference complete genome of 2603V/R, the first fully sequenced GBS strain, belonging to the Clonal Complex 19 (CC19) [33].

**Fig 1:**
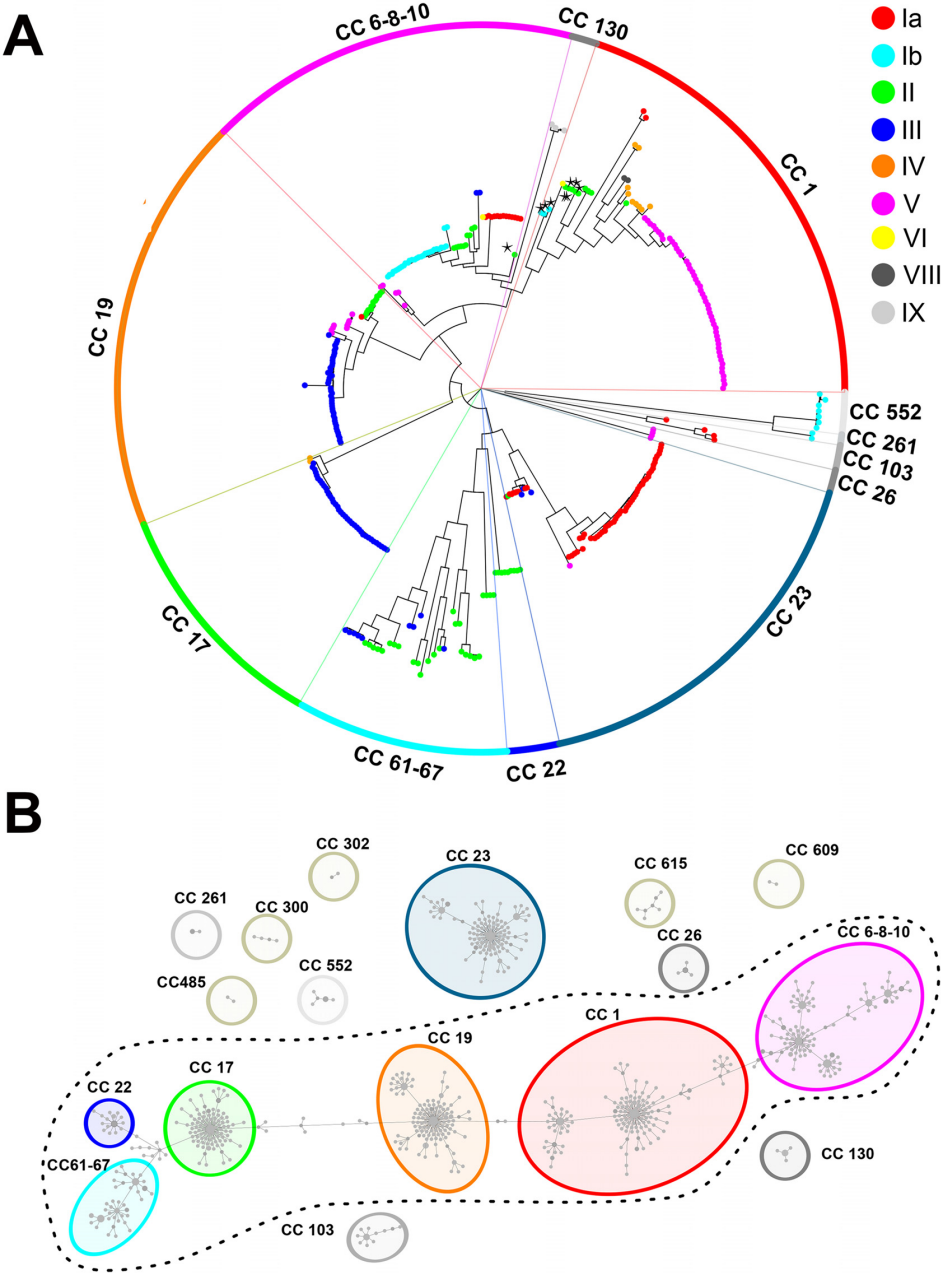
GBS SNP-based and MST-based phylogenetic trees. (A) SNP-based Neighbor-joining phylogenetic tree. The tree was generated using 14,092 polymorphic sites extracted from the alignment of 0.42 Mbp non-duplicated core regions shared by all 373 strains aligned to the reference strain 2603 V/R. CCs assigned to the 12 major clusters are indicated in colored ribbons. Dots represent single strains and are colored according to their capsular genotype. Asterisks indicate strains where the CC assigned by MLST (CC-1 or CC-6-8-10) differs from that assigned to strains belonging to the same SNP clade. (B) Minimum Multilocus Sequence Typing spanning tree of GBS strains. Each node represents one ST and STs differing by only one allele are connected by a line. Node dimensions refer to the relative number of strains belonging to each ST. Colored dots represent the assigned 17 CCs after refinement based on the SNP analysis. CCs included in the dotted line circle are linked by transition STs.

A neighbor joining phylogenetic tree constructed on the basis of the 14,092 detected SNPs is shown in Fig 1A. The tree appeared constituted by 6 major clades containing from 34 to 69 isolates, plus 6 smaller lineages comprising 2 to 9 strains. Two random strains belonging to separate clades differed on average by 2,208 SNPs, while the average number of SNPs within each clade was 471. The capsular genotypes of the 373 strains included in this analysis are indicated by dots in different colors in Fig 1A. As shown, strains belonging to the same capsular genotype were mostly grouped in monophyletic branches. Yet there were few exceptions of “misplaced” strains indicative of capsular switching (at least 10 strains, of which 5 NT).

We next assigned Sequence Types (STs) to the 128 internally sequenced isolates (S2 Table) and generated a Minimum Spanning Tree (MST) by integrating these data with the STs already present in the GBS MLST database (http://pubmlst.org/sagalactiae). The obtained MST tree (Fig 1B) contained 706 previously described STs, plus 12 STs extracted from the newly sequenced genomes. Similar to what was previously observed by Martins *et al*., [34], a clear separation between CCs, *i*.*e*. clusters of related STs differing by only one allele and associated by a founder ST, was not achievable due to the presence of a number of transition STs linking more than one previously described CC. As a result, strictly speaking most STs constituted a single large group (dotted line in Fig 1B).

A better discrimination between CCs belonging to different population lineages was achieved by taking advantage of the conducted genome-wide SNP analysis. This integrated MLST/SNP approach allowed assigning most GBS STs to 17 CCs (Fig 1B and S2 Table), 6 of which interconnected by transition STs and 11 independent clusters. Twelve of these 17 CCs correspond to the 12 clades defined by the SNP-based tree (Fig 1A).

**Fig 2:**
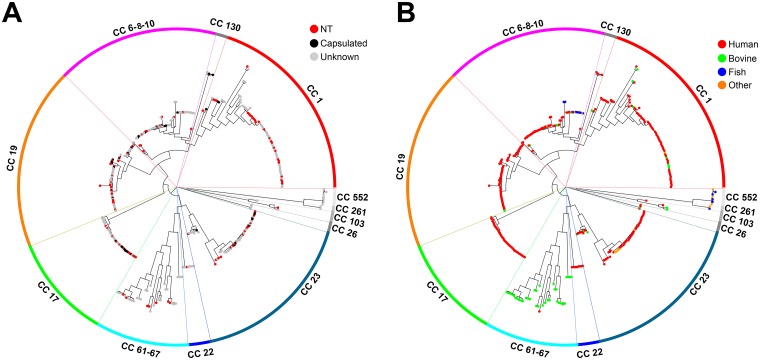
GBS SNP-based Neighbor-joining phylogenetic trees highlighting GBS hosts and NT strains. (A) Phylogenetic tree where non-typeable or capsulated phenotypes are indicated by colored dots. (B) Phylogenetic tree where strain host origin is indicated by colored dots.

Concerning the distribution of non-encapsulated strains in the GBS population, they were found in all the main lineages of the obtained SNP phylogenetic tree (Fig 2A). This random distribution suggests that genetic mutation events leading to capsule loss occur randomly in the GBS population. Interestingly, we found 11 capsular genotype III human NTs belonging to CC17, a hypervirulent CC frequently associated to late-onset neonatal disease [35].

Fig 2B displays the same SNP-based tree as in Figs 1A and 2A, but highlighting each GBS strain according to its host species. As shown, human strains were found in all clades, while strains isolated from frog, dog, dolphin and fish species clustered in the clades corresponding to clonal complexes CC23, CC6-8-10, CC552 and CC261. Few of the bovine strains were found in clades mainly containing human isolates, while most of them belonged to a highly divergent separate clade (average of 1,304 SNPs) corresponding to the CC61–67. This bovine CC appeared phylogenetically related to the human CC22, but not to the neonatal hypervirulent CC17 as previously predicted on the basis of MLST analysis only [35].

Taking advantage of 123 fully annotated internally sequenced genomes, we sought to compare the diversity between GBS isolates of bovine (n = 10) and human (n = 113) origin and between encapsulated (n = 32 strains) and NT (n = 91) strains at the level of the full pan-genome, i.e including both core and accessory genes. To this aim, we first extracted all the coding sequences from these genomes and the corresponding proteins were clustered by the CD-HIT algorithm (http://weizhong-lab.ucsd.edu/cd-hit/) using 70% amino acid identity and 70% query coverage as cut-off. The analysis yielded 6790 clusters each containing orthologous proteins, of which 1435 were found in at least 95% of the analyzed genomes while the remaining 5355 were considered accessory clusters.

To assess diversity in the bovine CC61–67 and in the five main human CCs 1, 6-8-10, 17, 19 and 23, we performed pairwise comparisons for every unique combination of two strains within each of these CCs. The distance between pairwise compared strains was defined as the semi-sum of the number of protein clusters exclusively identified in either of the two strains. As shown in S3A Fig, the average distance between pairwise strains was about two fold higher within the CC61–67 than within each of the five human CCs (P<0.0001), consistent with the higher variability of the bovine CC already revealed by SNP analysis of the core genome (Fig 2B).

We next compared the diversity between NT and encapsulated strains within each of the human CCs 17, 23, 19, 6-8-10 (at least 9 NT and 7 encapsulated isolates in each CC). The corresponding pairwise distances are, shown in S3B Fig, and indicated a slightly higher diversity for the NT strains compared to their encapsulated counterparts of the same CC (1.2–1.4 fold, P<0.01).

### Identification of genetic events leading to loss of capsule expression

To identify the type of genetic alterations that most often lead to capsule loss in GBS, we extracted the DNA sequences corresponding to the *cps* locus and its 5’ flanking region (20 kbp in total) from the genomes of the 96 NT sequenced strains.

The extracted DNA sequences were aligned with the internally derived *cps* reference sequences from encapsulated strains and with those present in public databases. The analysis confirmed the capsular genotypes of all 96 NT strains predicted by PCR analysis, and allowed detecting genetic alterations potentially responsible for the NT phenotype.

We found that all 96 NT strains differed from their reference counterparts by at least one nucleotide, either in the promoter region or in the *cps* ORFs. More specifically, 89 strains contained single or multiple genetic alterations representing a total of 125 different mutations that are likely to prevent capsule expression. These included deletions affecting multiple *cps* genes (range 80–9,059 bp), insertions of large transposable elements, point mutations in the -10 promoter region, and insertions/deletions (indels, range 2–253 bp) or point mutations resulting in frame-shifts and/or in premature stop codons in one of the *cps* genes. The remaining 7 strains contained only non-synonymous single nucleotide polymorphisms that could not be unambiguously associated to gene inactivation or impaired transcription. These missense mutations are described in detail in a separate section below.

**Fig 3:**
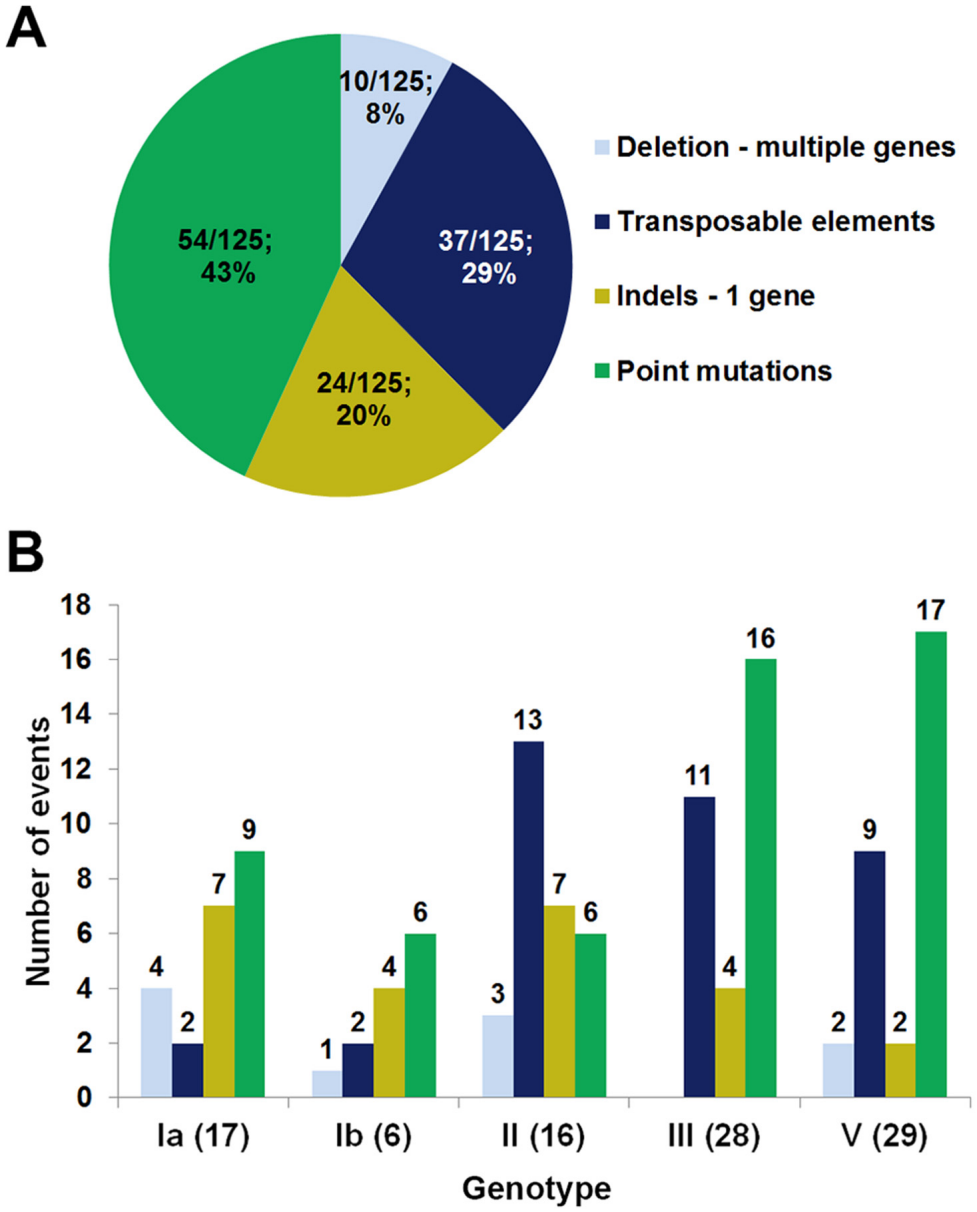
Relative frequency of the different types of mutations possibly responsible for the lack of capsule expression in GBS. (A) Distribution of 126 genetic alteration events detected in 89 isolates. (B) Number of strains bearing each of the *cps* mutation types among to the five selected capsular genotypes.

The relative frequencies of the 125 different types of detected genetic events potentially responsible for the lack of capsule expression are reported in Fig 3A. Point mutations causing premature stop codons were the most prevalent, followed by transpositions, deletions affecting more than one *cps* gene and indels targeting a single gene. The relative frequencies of the different types of genetic alterations among the five investigated capsular genotypes are shown in Fig 3B.

As reported in S2 Table, 62 isolates showed only one single alteration, while more than one inactivating mutation was present simultaneously in 27 isolates.

Of note, 30 strains contained non-unique mutations that were found in more than one isolate, either alone or in combination with other mutations. In particular, 10 single point mutations were encountered in 2 to 8 different strains. Additional genomic comparison confirmed that strains that carried the same *cps* mutation were not identical in the DNA regions outside the capsular locus.

### Target genes of *cps* inactivation mutations

We next investigated the frequency of genetic alterations possibly causing inactivation of capsule expression in each of the genes and the promoter of the *cps* operon.

This DNA region is composed by 16–18 genes in the different GBS serotypes [36]. The 5’ *cpsABCD* genes are predicted to be involved in the regulation of capsule synthesis; the central region from *cpsE* to *cpsL* encodes the enzymes responsible for the synthesis, transport, and polymerization of the polysaccharide repeating units; finally, *neuBCDA* genes are responsible for the synthesis of the activated sialic acid, a sugar component present in all GBS capsular polysaccharides [37].

**Fig 4:**
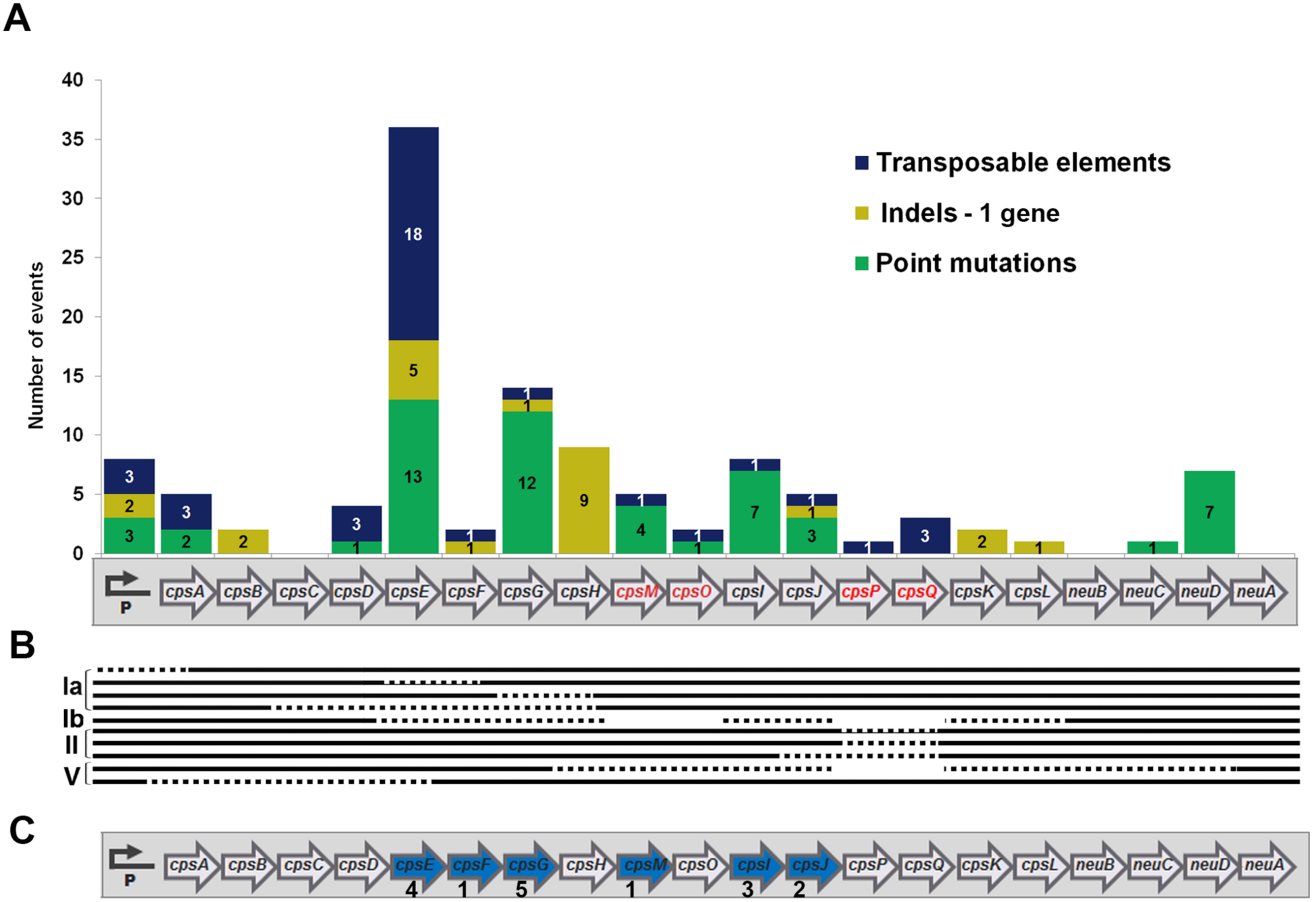
Different kinds of genetic alterations detected in the GBS *cps* operon. (A) Distribution of 37 transposable elements (blue), 24 insertion or deletion (indels) targeting a single gene (light green) and 54 point mutations (dark green) scattered across the *cps* operon. Genes that are missing in one or more capsular types are shown with red font color in the operon diagram. (B) Deletions comprising more than a single gene are indicated with dotted lines; empty spaces represent the absence of *cps* genes in a specific capsular genotype. (C) Gene targets of capsule inactivation mutations that appeared as single events (blue arrows); the figures below the target genes indicate the number of strains presenting the individual mutation.

The number of point mutations, indels, and transposable elements detected along the operon are indicated in Fig 4A. As shown, the *cps* promoter region and all *cps* genes except *neuA* and *cpsC*, were targets of at least one kind of genetic alteration.

Overall, we found 55 point mutation events across 10 *cps* genes or the promoter, and the most frequent targets were *cpsE* and *cpsG*, encoding the first and second enzymes in the synthesis pathway of the polysaccharide repeating unit. Remarkably, we did not find any NT strains harboring capsule inactivating point mutations in *cpsA*, *cpsB*, *cpsC* or *cpsD*.

Twenty-four indels were detected in 8 *cps* genes or the promoter, and most of these were found in the *cpsH*. This gene is homologous to wzy from *E*. *coli* and from *S*. *pneumoniae*, presumably encoding the polymerases that link repeating units via glycosidic linkages to form the long polysaccharide chains [37,38].

Ten isolates contained deletions affecting more than one gene, while 37 transposable elements were present in 32 isolates (Fig 4B). The transposable elements present in the *cps* region of the different strains were classified according to the IS finder database nomenclature (http://www-is.biotoul.fr) [39]. In total, 9 different IS types were identified, 8 of which belonging to known IS families (Table 2). As shown, most IS were found in the *cpsE* gene, and the most frequently encountered IS type was IS1381 as previously reported for another NT GBS strain [14].

**Table 2:** Classification of the transposable elements identified in 96 sequenced non- encapsulated strains.

		*cps* target region(s)																				
IS type	IS family	Promoter	*cpsA*	*cpsB*	*cpsC*	*cpsD*	*cpsE*	*cpsF*	*cpsG*	*cpsH*	*cpsM*	*cpsO*	*cpsI*	*cpsJ*	*cpsP*	*cpsQ*	*cpsK*	*cpsL*	*neuB*	*neuC*	*neuD*	*neuA*
IS1381	IS5	3					10	1	1		1											
ISSa4	IS982						3						1	1		1						
IS1062 like	IS30						1															
ISSag11	IS256						2								1	1						
IS1548	ISAs1		3			3	1															
Unknown	Unknown						1															
ISSpo8	IS1595											1										
ISSpo8- like	IS1595						1															

Finally, sixteen of the 96 NT strains harbored only one single mutation resulting in a premature stop codon in the *cps* locus, and the target genes of these single mutations were *cpsE*, *F*, *G*, *I*, *J* and *cpsM* (Fig 4C).

### Mutations in amino acid residues essential for functional activity of CpsE

As shown above, 7 NT strains belonging to capsular genotypes Ia, II, III and V presented missense mutations for which the effect on capsule synthesis was unpredictable (Table 3). The genes carrying these mutations were compared with the corresponding genes from the 32 encapsulated reference strains and loci from genome draft sequences present in the NCBI database. All the missense mutations were unique to these seven NT isolates, and five of them targeted the *cpsE* gene. The CpsE portal enzyme of GBS capsule biosynthesis is highly conserved among the nine serotypes, with an amino acid identity ranging between 98.2% and 99.8%. BLAST analysis indicated that 4 of the detected mutations (R308G, R353I, P387S, R419K) were located in gene positions that were conserved in more than 95% of 500 examined CpsE homologs from a wide range of bacterial species.

**Table 3:** NT GBS strains presenting missense mutations and the results of *cpsE* complementation in same strains.

GBS isolate	Molecular Type	Target gene(s)	AA Mutation(s)	*cpsE* complementation
DK-PW-097	V	*cpsE; cpsM*	S236N; L94W	negative
325662	Ia	*cpsE*	R308G	positive
ES-PW-195	II	*cpsE*	R353I	positive
IT-PW-0094	III	*cpsE*	P301S; P387S	positive
BE-PW-101	V	*cpsE*	R419K	positive
SH2515	V	*cpsH; cpsM*	T178I; S195F	n.a.
SH3115	III	*cpsG*	G73V	n.a.

The nucleotide and amino acid changes in the isolates were based on the encapsulated 2603 V/R reference genome sequence. AA, single letter amino acid designation; del, deletion of the nucleotide; n.a. not applicable.

**Fig 5:**
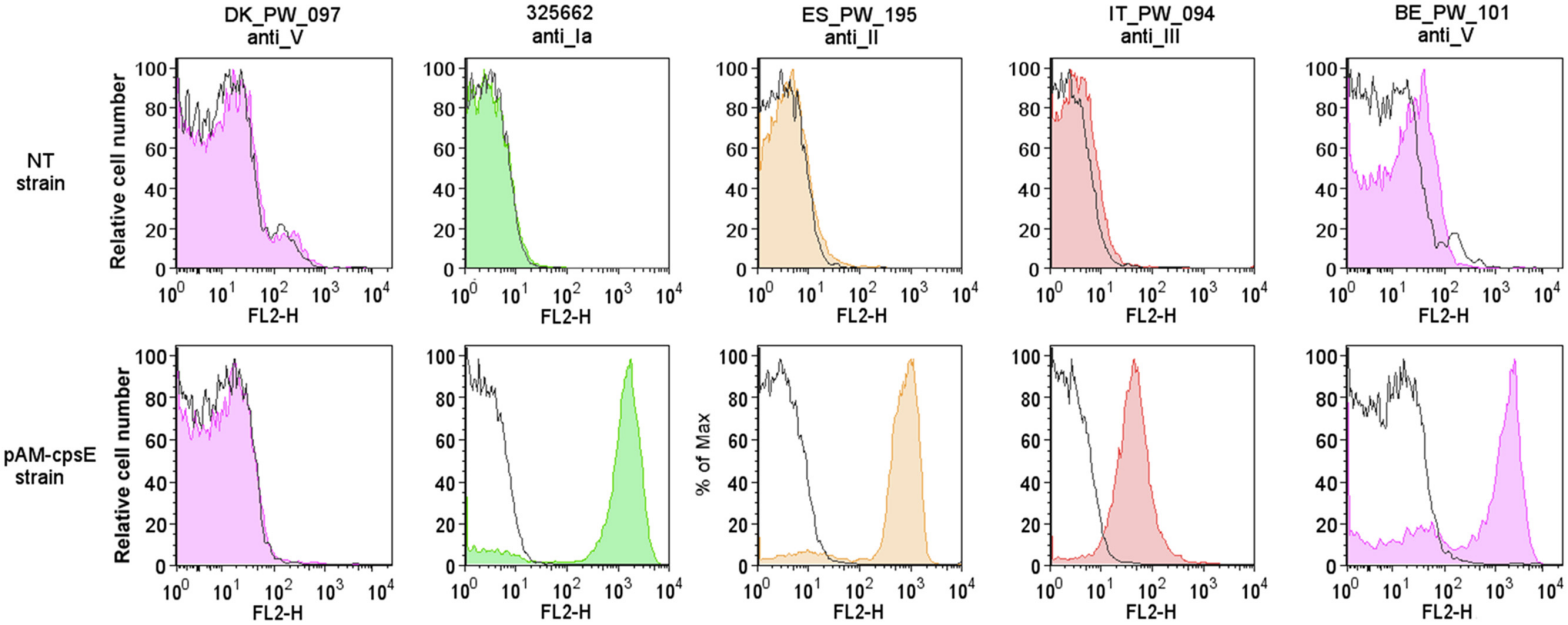
Complementation of *cpsE* missense mutations in selected NT GBS strains. Flow cytometry analysis of NT GBS strains carrying missense mutations in *cpsE* and of their counterpart after transformation with pAM-*cpsE*. Bacteria were incubated with mouse monoclonal antibodies specific for the five capsular polysaccharides, and then treated with labeled secondary antibodies. Fluorescence after incubation with the secondary antibody alone is indicated by empty histograms, while staining after treatment with type the specific antibodies is shown by the colored histograms.

We investigated whether the CpsE amino acid substitutions were sufficient to account for capsule loss. The plasmid pAM-*cpsE* containing a wild type *cpsE* Ia gene sequence was generated and transformed into the five different NT strains harboring mutations in *cpsE*. The transformed bacteria were tested for their capacity to synthesize a capsular polysaccharide by latex agglutination and by flow-cytometry assays. As shown in Fig 5, CpsE overexpression restored capsular synthesis in the 4 strains carrying exclusively one missense mutation in the *cpsE* gene. The results show that the highly conserved amino acid residues R308, R353, P387, R419 are essential for enzyme activity. One of the five mutations could not be complemented, possibly due to a concomitant missense mutation in the *cpsM* gene.

### Genetic events that abolish transcription of the *cps* operon

**Fig 6:**
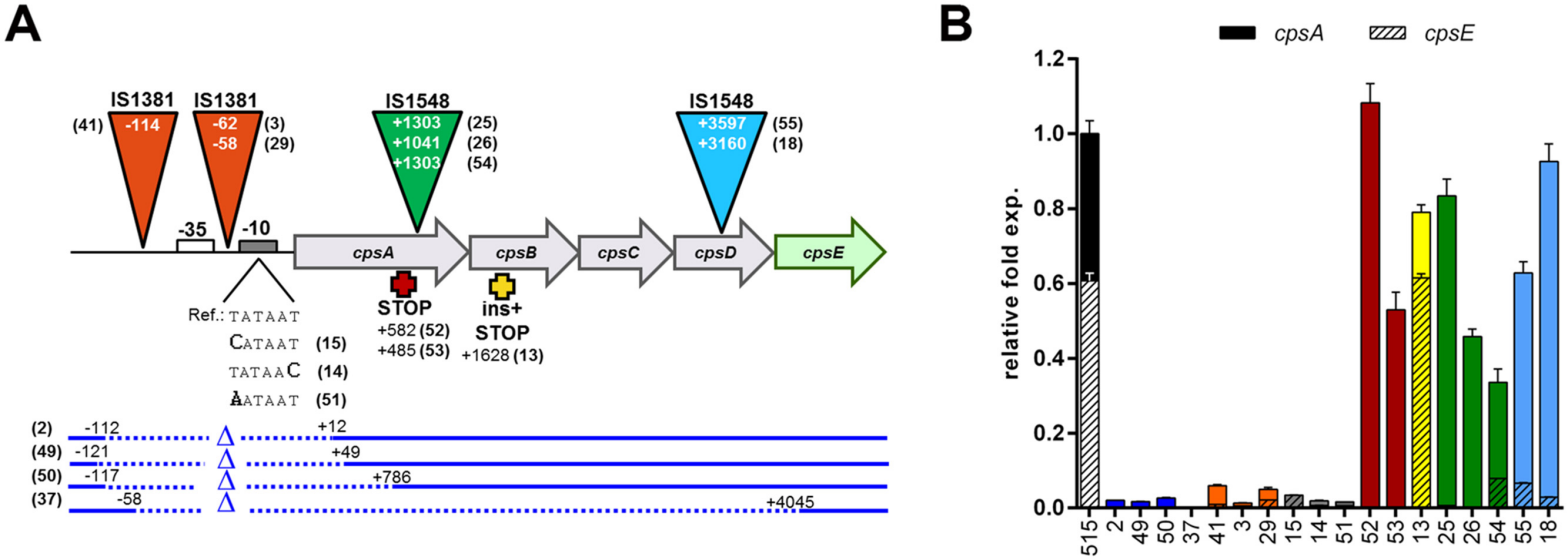
Analysis of the influence of genetic alterations on the transcription of the *cps* operon in NT isolates. (A) Mutations in the *cps* promoter or *cpsA-D* detected in 18 NT strains. Their positions are indicated as +/- numbers in relation to the first base pair of the *cpsA* coding sequence. ISs are represented by colored triangles; point mutations in the -10 sequence are marked in bold; deleted regions are represented by dotted lines and stop codons by crosses. Numbers within parentheses identify the strains reported in the x-axis of panel B. (B) Transcription of the *cps* operon in the 18 NT strains measured with primers cpsAup-F/R (filled bars) and to cpsE-F/R (hatched bars). The relative fold expression for each strain was estimated in comparison to the expression of *cpsA* in strain 515. For strains 25, 26, 52 and 53 the transcript in *cpsE* gene was not determined.

The transcription of the cps operon is a prerequisite for capsule production. Consequently, mutations leading to insufficient or abolished transcription were expected to result in a NT phenotype. We used qRT-PCR to compare the transcription of the cps operon in a subset of 48 NT and 6 encapsulated reference isolates (strains 1–48 in S2 Fig). The operon is transcribed from the promoter region upstream of its first gene *cpsA* [40], therefore, two primers annealing to the 3’ region of *cpsA* were used (see Methods section). As shown in S2 Fig, the *cpsA* transcript amount for most strains was normally distributed between 0.4–1.42 fold compared to the encapsulated strain GBS515. However, 9 NTs were devoid of any detectable *cpsA* transcript and, notably, all of them presented point mutations in the promoter region or IS either in the promoter or the first four genes of the operon (Fig 6A). On the basis of the above observations, we subsequently selected for further transcription analysis all the NT isolates presenting genetic alterations in the *cps* promoter region or the *cpsA*, *B*, *C*, *D* genes. Eighteen out of 96 NT isolates, including the 9 above described strains lacking the *cpsA* transcript, contained different kinds of mutations in this region. These alterations comprised IS elements inserted in the vicinity of the Shine-Dalgarno sequence, point mutations in the -10 box, large deletions comprising the *cps* promoter, IS elements in the *cpsA* and *cpsD* coding regions, and point mutations or indels leading to stop codons in *cpsA* and *cpsB* (Fig 6A).

Therefore, all 18 isolates were then analyzed by qRT-PCR using primers annealing to the *cpsE* gene, and to the 5’ region of the *cpsA* gene (upstream the different IS elements identified in this gene). As shown in Fig 6 B, all the mutations present in the promoter region completely abolished transcription. In the strains containing IS elements in *cpsA* or in *cpsD* we found transcripts upstream the transposable element, but not downstream of this insertion. Finally, genetic alterations leading to premature stop codons in *cpsA* or *in cpsB* appeared to have limited or no effect, indicating that these genes are not essential for *cps* transcriptional regulation.

## Discussion

The sialic acid containing capsular polysaccharide that surrounds GBS is essential for bacterial survival in animal models of infection. Furthermore, vaccines targeting this antigen are in development phase. The occurrence of non-encapsulated GBS strains devoid of this important protective “shield” is therefore difficult to explain and could be of concern in GBS vaccinology.

In the present study we confirmed the complete absence of capsule expression in 96 well-characterized GBS isolates, and 27 of these strains were obtained from patients suffering from invasive disease.

In principle one cannot exclude that in some of the cases the NT phenotype could have arisen in an already infected patient, or after isolation of the microorganism during *in vitro* cultivation. However, 11 of the 27 NT strains causing invasive disease harbored more than one mutation possibly leading to capsule inactivation, and it appears highly unlikely that in all these strains more than one independent mutation event could occur during or after infection. We show that the NT phenotype occurs randomly in all lineages of the GBS population and that it is mainly associated with genetic alterations in the *cps* operon. Capsule loss can also occur among hypervirulent CC17 strains, and some of the resulting non-encapsulated isolates are still able to cause invasive disease in humans.

The observed random distribution of non-encapsulated isolates in the GBS population differs from the distribution observed for *S*. *pneumoniae*, where the large majority of NTs cluster in few CCs that consist almost exclusively of non-encapsulated strains (http://pubmlst.org/spneumoniae/). Indeed, a comprehensive epidemiological study on pneumococcal carriage revealed a high frequency of NT strains mostly belonging to one single phylogenetic lineage [41]. This NT lineage showed the highest rates of receipt and donation of recombined DNA fragments among pneumococcal clades, and the authors proposed that carriage NT strains could be a major reservoir of genetic diversity in this species [42].

The NT phenotype is much less frequent among GBS strains obtained from human carriers compared to the pneumococci, suggesting the absence of selective advantage for human colonization. However, the high frequency (up to 77%) of NTs among isolates obtained from bovine mastitis could reflect a high degree of fitness for colonization of the udder epithelia. Alternatively, less efficient complement deposition on the surface of GBS present in bovine milk compared to human blood [43] could make the capsule shield less necessary.

Different types of genetic alterations appear to be responsible for the inactivation of capsule synthesis in GBS such as point mutations, deletions targeting single or more than one cps gene, short insertions and presence of transposable elements. The three main gene targets of these mutations were *cpsE*, *cpsG* and *cpsH*. Mutations introducing stop codons in these three genes in type Ia background had been shown to result in complete capsule loss [14,36], and our results confirm the essential role of the corresponding enzymes in the synthesis of the GBS capsule.

The CpsE enzyme catalyzes the addition of glucose-1-phosphate to undecaprenyl carrier of the bacterial membrane as first reaction in the synthesis of the polysaccharide repeating unit [38]. CpsE overexpression restored capsular synthesis in the 4 strains carrying exclusively one missense mutation in the *cpsE* gene, allowing the identification of amino acid residues that are essential for enzyme activity. Moreover, expression of a single enzyme variant rescued biosynthesis of different capsular types, confirming that serotype specificity does not depend on CpsE.

On the basis of its homology with a well-characterized galactosyl transferase from *S*. *pneumoniae*, CpsG was predicted to attach galactose to undecaprenyl-glucose during the second step of polysaccharide repeating unit biosynthesis [37]. It is tempting to speculate that inactivating mutations in the enzymes responsible for the early steps of capsule biosynthesis could prevent unnecessary energy expenditure and potential sequestration of monosaccharides.

We also detected non-encapsulated strains that contained only one single mutation resulting in a premature stop codon in *cpsF*, *G*, *I*, *J* and *cpsM*. The data suggest that, in addition to the above mentioned *cpsE*, *G* and *H*, these genes are essential for GBS capsule expression.

In agreement with findings from Cieslewicz *et al*., we did not find any NT strains that harbored single capsule inactivation mutations in *cpsA*, *cpsB*, *cpsC* or *cpsD* [36]. In fact, these authors demonstrated that out of five genes examined by in-frame deletion mutations (*cpsA*, *cpsB*, *cpsC*, *cpsD* and *cpsE*), only a disruption of *cpsE* resulted in the non-encapsulated phenotype. It was therefore suggested that *cpsA*-*D* are not required for biosynthesis of the repeating unit, but rather direct the coordinated polymerization and export of the polysaccharide [36]. The authors also observed a decreased transcription in the *cpsA* knockout mutant. According to our observations, genetic alterations leading to premature stop codons in *cpsA* or in *cpsB* appeared to have limited or no effect, indicating that these genes are not essential for *cps* transcriptional regulation.

Genome wide analysis conducted on both NT and encapsulated strains provided new insights into the evolution of the GBS species and its adaptation to cause human disease. The phylogenetic tree obtained by integrated SNP-based analysis of the 128 sequenced isolates plus the 245 publicly available genomes appeared constituted by 6 major clades, plus 6 smaller lineages comprising a low number of strains. The tree was consistent with the one recently reported by Da Cunha *et al*. [44]. The authors noted that human strains belonging to the same phylogenetic clade contained a limited number of polymorphisms, and proposed that this clonal pattern could be the result of recent global spreading of few successful GBS clones having acquired a conjugative element conferring resistance to tetracycline after the introduction of this antibiotic in 1948. Our analysis confirmed very low variability within clades containing mainly human strains (from 50 SNPs for CC17 to 601 SNPs for CC6-8-10). However, it revealed a higher degree of diversity in the bovine population (average of 1,304 SNPs within the CC61–67). This higher diversity was also confirmed by extending the analysis to the accessory genome, and is consistent with previous observations by Sorensen *et al*. [45], presumably reflecting a longer evolutionary history of this bovine CC. Evidence that some of the bovine strains can be found in clades mainly containing human isolates is compatible with GBS transmission between the two hosts. Previous studies identified the *lac* genes involved in lactose fermentation and the *scpB* gene encoding the C5a peptidase as more frequently found in strains of bovine versus human origin and viceversa, respectively [46,47]. We interrogated the 372 genomes by a BLAST search, and found the *lac* genes in all the 53 available bovine GBS genomes, and only in 8 human strains (all belonging either to CC1 or CC23). The *scpB* gene was instead found in 233 out of 291 human and 6 out of 53 bovine isolates. Remarkably, the *tet M* gene conferring Tetracycline resistance was found in 216 out of 291 human strains and only in 6 out of 53 strains from bovine origin (none of which belonging to CC61–67), consistent with the above mentioned hypothesis according to which most human GBS strains would have arisen from few clones having acquired a conjugative element bearing *tetM*.

Finally, the slightly higher diversity detected in the NT accessory genomes compared to the encapsulated strains might suggest a higher uptake of foreign genetic elements in the first group, although this will need to be confirmed with more in depth analysis. Further mining into the now available large number of genomes is indeed expected to provide new important hints on GBS host adaptation and pathogenesis mechanisms.

The strategy used by non-encapsulated GBS strains to evade the innate immune system remains unknown. Further research in this field will be crucial for the development of new preventive and therapeutic initiatives against this relevant human and animal pathogen.

## Supporting Information

S1 Fig Flow cytometry analysis of representative encapsulated and non-encapsulated GBS isolates. Encapsulated (A) or non-encapsulated bacteria (B) were stained with mouse monoclonal antibodies anti-Ia, Ib, II, III and V, and labeled with R-Phycoerythrin conjugated secondary antibodies. Bacterial staining obtained with pre-immune mouse serum is indicated by black histograms, and staining with type specific antibodies is shown by colored histograms. Empty histograms indicate the signals obtained with antibodies against heterologous capsular types.
(TIF)

S2 Fig Relative transcription of *cpsA* in NT and encapsulated GBS strains. Transcript levels in NT (grey) and encapsulated strains (black) obtained using primers cpsA-F/R were compared to those of the housekeeping gene *gyrA* by qRT-PCR. The relative fold expression for each strain was in comparison with strain 515. Columns show results from three independent growth replicates, each analyzed performed in technical triplicates. Error bars represent standard deviations.
(TIF)

S3 Fig Diversity within major human and cattle GBS CCs. Pairwise comparisons were performed for every unique combination of two strains within the indicated CCs (x-axis). The distances between strain pairs (y-axis) were defined as the semi-sum of the number of unique protein classes identified in the two compared strains. In the box-and-whiskers plots, the box represents the interquartile range and the whiskers extend from the minimum to the maximum distances; the median distances are represented by a horizontal line and the average distances by a cross; statistical differences between groups were calculated by the two-tailed Mann Whitney test. (**A)** Pairwise distances between CCs. Significant differences were detected between the bovine CC61–67 and the human CCs1, 6-8-10, 17, 19 and 23 (P < 0,0001), as well as between CCs 6-8-10 or 19 and CCs 1, 17 or 23 (P < 0.01). (**B)** Pairwise distances between NT and encapsulated strains belonging to CCs 6-8-10, 17, 19 and 23. Significant differences between NT and encapsulated strains were detected in all four CCs (P<0.01)
(TIF)

S1 Table Primers used for qRT-PCR analysis of *cps* operon transcription. (PDF)

S2 Table Detailed information on the 96 NT GBS strains and 32 non-encapsulated strains selected for genome sequencing. DEL: Deletion of multiple genes; PM: Point mutations leading to stop codons; IND: Insertions/Deletions involving 1 gene and leading to stop codons; MIS: Missense point mutations; LOD: Late Onset Disease; EOD: Early Onset Disease; ID: Invasive Disease; ST: Sequence type; CC: Clonal Complex.
(PDF)
