# Supplementary figures and images for: Genomic Analysis Reveals the Molecular Basis for Capsule Loss in the Group B Streptococcus Population
Source: PLoS One. 2015 May 6;10(5):e0125985. doi: 10.1371/journal.pone.0125985 (PMC4422693; doi:10.1371/journal.pone.0125985)

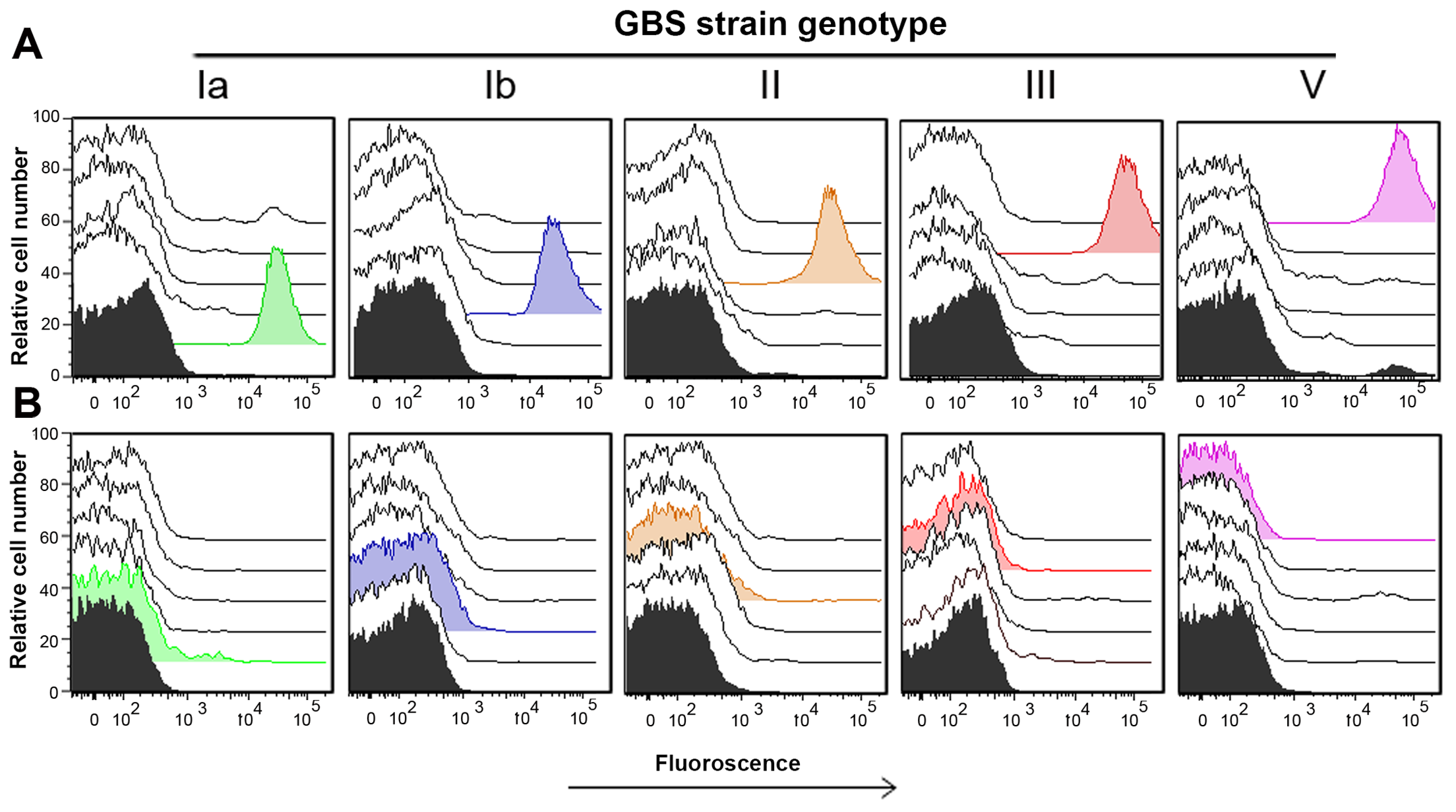

Supplement: S1 Fig — Encapsulated (A) or non-encapsulated bacteria (B) were stained with mouse monoclonal antibodies anti-Ia, Ib, II, III and V, and labeled with R-Phycoerythrin conjugated secondary antibodies. Bacterial staining obtained with pre-immune mouse serum is indicated by black histograms, and staining with type specific antibodies is shown by colored histograms. Empty histograms indicate the signals obtained with antibodies against heterologous capsular types. (TIF) [file pone.0125985.s001.tif]

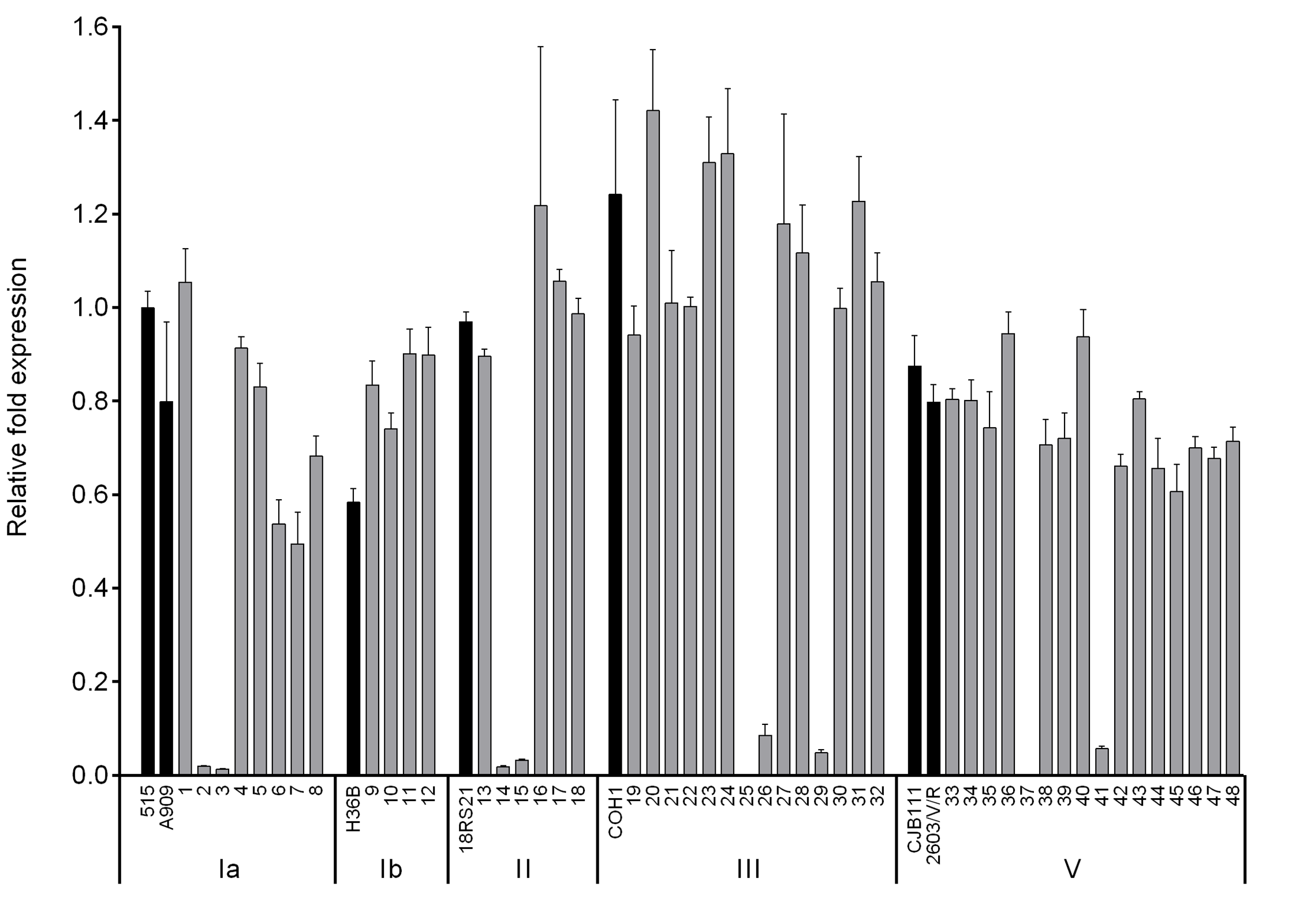

Supplement: S2 Fig — Transcript levels in NT (grey) and encapsulated strains (black) obtained using primers cpsA-F/R were compared to those of the housekeeping gene gyrA by qRT-PCR. The relative fold expression for each strain was in comparison with strain 515. Columns show results from three independent growth replicates, each analyzed performed in technical triplicates. Error bars represent standard deviations. (TIF) [file pone.0125985.s002.tif]

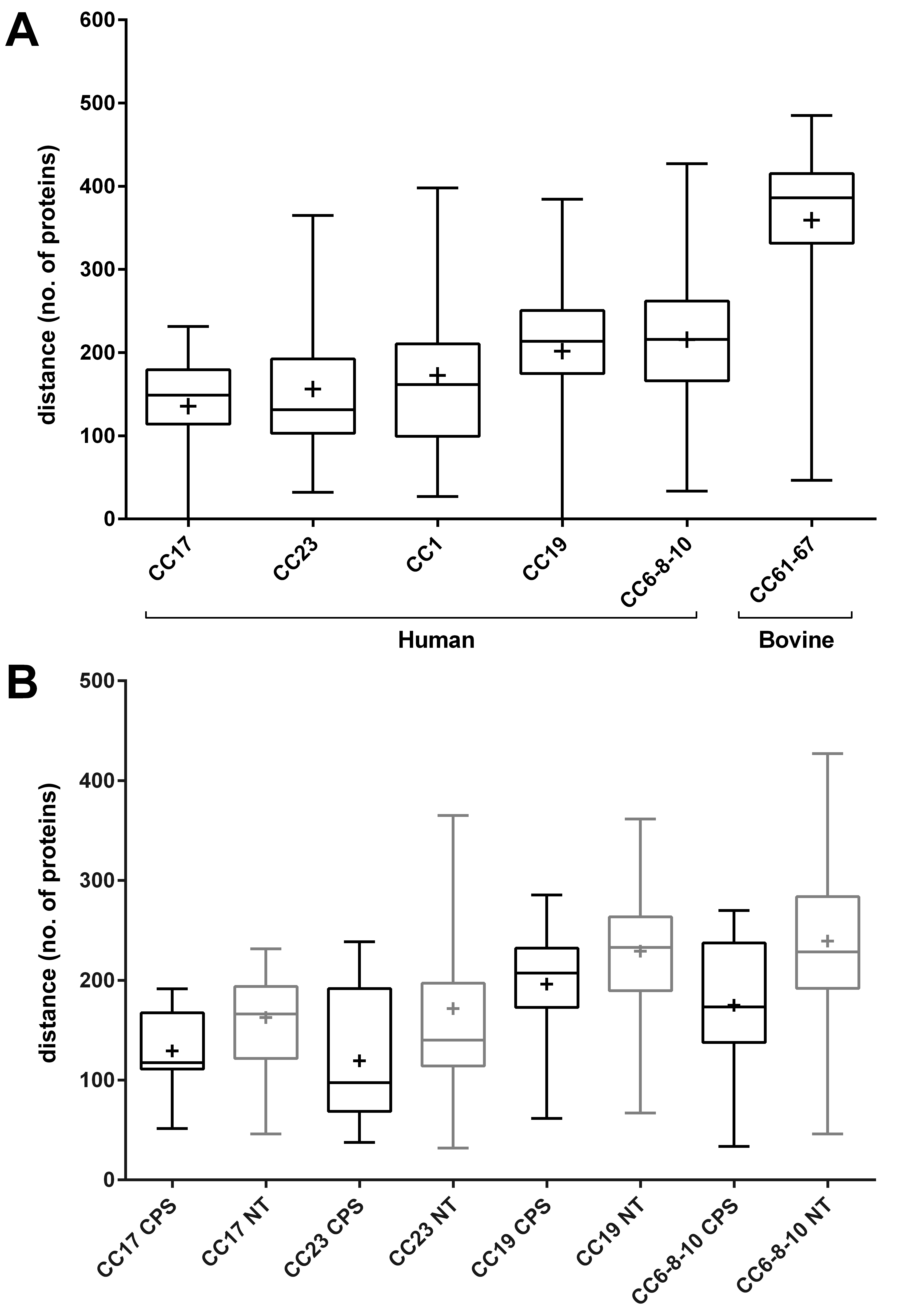

Supplement: S3 Fig — Pairwise comparisons were performed for every unique combination of two strains within the indicated CCs (x-axis). The distances between strain pairs (y-axis) were defined as the semi-sum of the number of unique protein classes identified in the two compared strains. In the box-and-whiskers plots, the box represents the interquartile range and the whiskers extend from the minimum to the maximum distances; the median distances are represented by a horizontal line and the average distances by a cross; statistical differences between groups were calculated by the two-tailed Mann Whitney test. (A) Pairwise distances between CCs. Significant differences were detected between the bovine CC61–67 and the human CCs1, 6-8-10, 17, 19 and 23 (P < 0,0001), as well as between CCs 6-8-10 or 19 and CCs 1, 17 or 23 (P < 0.01). (B) Pairwise distances between NT and encapsulated strains belonging to CCs 6-8-10, 17, 19 and 23. Significant differences between NT and encapsulated strains were detected in all four CCs (P<0.01) (TIF) [file pone.0125985.s003.tif]
