# Supplementary material for: Genomic Analysis Reveals the Molecular Basis for Capsule Loss in the Group B Streptococcus Population
Source: PLoS One. 2015 May 6;10(5):e0125985. doi: 10.1371/journal.pone.0125985 (PMC4422693; doi:10.1371/journal.pone.0125985)
Supplement: S1 Table — (PDF) [file pone.0125985.s004.pdf]

S1 Table. Primers used for qRT-PCR analysis of *cps* operon transcription

Primer name	Sequence 5'- 3'
gyrA-F	AGGTTTACTTGTGGCGCTTG
gyrA-R	TCTGCTTGAGCAATGGTGTC
cpsA-F	TCAACTGGACAACGCTTCAC
cpsA-R	AAGTTGAGCTCCTGGCATTG
cpsAup-F	TGAAAACCTTTACGACATCAACC
cpsAup-R	AAACTGTCACTCCTCCCAAGAG
cpsE-F	TGCTCATATGTGGCATTGTG
cpsE-R	AGAAAAGATAGCCGGTCCAC
