# Supplementary material for: Genomic Analysis Reveals the Molecular Basis for Capsule Loss in the Group B Streptococcus Population
Source: PLoS One. 2015 May 6;10(5):e0125985. doi: 10.1371/journal.pone.0125985 (PMC4422693; doi:10.1371/journal.pone.0125985)
Supplement: S2 Table — DEL: Deletion of multiple genes; PM: Point mutations leading to stop codons; IND: Insertions/Deletions involving 1 gene and leading to stop codons; MIS: Missense point mutations; LOD: Late Onset Disease; EOD: Early Onset Disease; ID: Invasive Disease; ST: Sequence type; CC: Clonal Complex. (PDF) [file pone.0125985.s005.pdf]

S2 Table. Detailed information on the 96 NT GBS strains and 32 non-encapsulated strains selected for genome sequencing

Strain Code	Host	Source	Capsular Genotype	Capsular Phenotype	Target genes of genetic alterations	Type of genetic alterations	ST	CC
IT-PW-0085	Human	Colonization	Ia	nt	*cpsC-cpsD-cpsE-cpsF-cpsG-cpsH*	DEL	23	23
DK-PW-070	Human	Colonization	Ib	nt	*cpsE-cpsF-cpsG-cpsH-cpsI-cpsJ-cpsK-cpsL*	DEL	8	6_8_10
312754	Human	Colonization	Ia	nt	*cpsG-cpsH*	DEL	24	23
GB-PW-075	Human	Colonization	V	nt	*cpsH-cpsM-cpsO-cpsI-cpsJ-cpsK-cpsL-neuB-neuC-neuD*	DEL	1	1
GB-PW-094	Human	Colonization	Ia	nt	promoter-*cpsA*	DEL	24	23
SH1287	Human	Adult ID	Ia	nt	*cpsE-cpsF, cpsL*	DEL, IND	23	23
DE-PW-406	Human	Colonization	V	nt	*cpsA-cpsB-cpsC-cpsD, cpsE*	DEL, IS1381	1	1
GB-PW-035	Human	Colonization	V	nt	*cpsE*	IND	19	19
IT-PW-0057	Human	Colonization	V	nt	*cpsE*	IND	1	1
DK-B-USS-084	Bovine	Mastitis	Ia	nt	*cpsE*	IND	103	103
BE-PW-051	Human	Colonization	III	nt	*cpsF*	IND	19	19
ML41151	Human	Colonization	III	nt	*cpsH*	IND	328	19
SH3683	Human	Adult ID	Ia	nt	*cpsJ*	IND	23	23
ES-PW-174	Human	Colonization	II	nt	*cpsK*	IND	88	23
DK-B-USS-155	Bovine	Mastitis	II	nt	promoter	IND	new	61_67
DK-B-USS-196	Bovine	Mastitis	II	nt	promoter	IND	415	61_67
DK-B-USS-204	Bovine	Mastitis	II	nt	*cpsE, cpsK*	IND, IND	new	61_67
379415	Human	Adult ID	III	nt	*cpsA, cpsG*	IND, PM	new	17
BG-PW-031	Human	Colonization	II	nt	*cpsB, cpsG*	IND, PM	28	19
309185	Human	Adult ID	Ia	nt	*cpsE, cpsH*	IND, PM	23	23
SH0655	Human	Adult ID	III	nt	*cpsE, cpsH*	IND, PM	286	19

AH20834	Human	Colonization	Ib	nt	*cpsE, cpsH*	IND, PM	8	6_8_10
GB-PW-081	Human	Colonization	Ib	nt	*cpsE, cpsH*	IND, PM	12	6_8_10
404288	Human	Adult ID	Ia	nt	*cpsE,neuD*	IND, PM	23	23
DK-B-USS-147	Bovine	Mastitis	II	nt	*cpsE, cpsJ, cpsP, cpsQ*	IS1062like, ISSag11	n.a.	61_67
ES-PW-181	Human	Colonization	III	nt	*cpsE*	IS1380	17	17
DE-PW-436	Human	Colonization	V	nt	*cpsE*	IS1381	1	1
DK-PW-089	Human	Colonization	V	nt	*cpsE*	IS1381	1	1
ES-PW-034	Human	Colonization	III	nt	*cpsE*	IS1381	17	17
ES-PW-088	Human	Colonization	III	nt	*cpsE*	IS1381	17	17
DK-B-USS-032	Bovine	Mastitis	V	nt	*cpsE*	IS1381	10	6_8_10
CZ-PW-143	Human	Colonization	V	nt	*cpsF*	IS1381	1	1
DE-PW-435	Human	Colonization	V	nt	*cpsM*	IS1381	new	1
BE-PW-095	Human	Colonization	III	nt	promoter	IS1381	19	19
DK-PW-092	Human	Colonization	V	nt	promoter	IS1381	new	6_8_10
IT-PW-0059	Human	Colonization	Ia	nt	promoter	IS1381	23	23
BE-PW-130	Human	Colonization	Ia	nt	*cpsE, cpsH*	IS1381, IND	23	23
B848	Human	Colonization	III	nt	*cpsA*	IS1548	new	19
ES-PW-118	Human	Colonization	III	nt	*cpsA*	IS1548	19	19
DK-PW-162	Human	Colonization	III	nt	*cpsD*	IS1548	19	19
GB-PW-067	Human	Colonization	II	nt	*cpsD*	IS1548	12	6_8_10
IMMI_409	Human	Colonization	III	nt	*cpsD*	IS1548	19	19
418136	Human	Colonization	III	nt	*cpsE*	IS1548	10	6_8_10
SH0396	Human	Adult ID	II	nt	*cpsE, cpsP-cpsQ*	ISSa4, DEL	12	6_8_10
SH0519	Human	Adult ID	II	nt	*cpsE, cpsP-cpsQ*	ISSa4, DEL	12	6_8_10
SH0986	Human	Adult ID	II	nt	*cpsE, cpsJ, cpsQ*	ISSa4, ISSa4, IS1381	12	6_8_10
DK-B-USS-215	Bovine	Mastitis	III	nt	*cpsE*	ISSag11	61	61_67
BE-PW-123	Human	Colonization	V	nt	*cpsE*	ISSpo8-like	1	1
SH2515	Human	Adult ID	V	nt	*cpsH, cpsM*	MIS	new	6_8_10

DK-PW-097	Human	Colonization	V	nt	*cpsE, cpsM*	MIS	518	1
IT-PW-0094	Human	Colonization	III	nt	*cpsE*	MIS	17	17
SH3115	Human	Adult ID	III	nt	*cpsG*	MIS	17	17
325662	Human	Colonization	Ia	nt	*cpsE*	MIS	new	23
BE-PW-101	Human	Colonization	V	nt	*cpsE*	MIS	1	1
ES-PW-195	Human	Colonization	II	nt	*cpsE*	MIS	new	22
ES-PW-121	Human	Colonization	Ia	Ia	none	none	23	23
090R/799	Human	Colonization	Ia	Ia	none	none	25	23
BE-PW-096	Human	Colonization	Ib	Ib	none	none	8	6_8_10
BE-PW-108	Human	Colonization	Ib	Ib	none	none	8	6_8_10
BE-PW-119	Human	Colonization	Ib	Ib	none	none	8	6_8_10
BG-PW-056	Human	Colonization	Ia	Ia	none	none	23	23
BG-PW-074	Human	Colonization	III	III	none	none	17	17
DK-PW-024	Human	Colonization	II	II	none	none	12	6_8_10
ES-PW-004	Human	Colonization	II	II	none	none	28	19
ES-PW-008	Human	Colonization	III	III	none	none	19	19
ES-PW-043	Human	Colonization	Ia	Ia	none	none	23	23
ES-PW-063	Human	Colonization	III	III	none	none	19	19
ES-PW-068	Human	Colonization	Ia	Ia	none	none	23	23
ES-PW-085	Human	Colonization	V	V	none	none	19	19
ES-PW-124	Human	Colonization	Ia	Ia	none	none	23	23
ES-PW-160	Human	Colonization	II	II	none	none	12	6_8_10
GB-PW-021	Human	Colonization	II	II	none	none	12	6_8_10
GB-PW-041	Human	Colonization	III	III	none	none	19	19
GB-PW-049	Human	Colonization	V	V	none	none	19	19
GB-PW-051	Human	Colonization	V	V	none	none	19	19
GB-PW-088	Human	Colonization	V	V	none	none	19	19
GB-PW-091	Human	Colonization	II	II	none	none	12	6_8_10

GB-PW-097	Human	Colonization	Ib	Ib	none	none	12	6_8_10
IT-PW-055	Human	Colonization	Ib	Ib	none	none	12	6_8_10
DE-NI-024	Human	LOD	III	III	none	none	17	17
DE-NI-035	Human	LOD	III	III	none	none	17	17
ES-NI-001	Human	EOD	III	III	none	none	17	17
ES-NI-003	Human	LOD	III	III	none	none	17	17
ES-NI-010	Human	LOD	III	III	none	none	17	17
IT-NI-001	Human	LOD	III	III	none	none	17	17
IT-NI-023	Human	LOD	Ia	Ia	none	none	23	23
IT-NI-025	Human	LOD	II	II	none	none	28	19
SH0248	Human	Adult ID	III	nt	*cpsE*	PM	17	17
SH0657	Human	Adult ID	V	nt	*cpsE*	PM	1	1
SH3601	Human	Adult ID	III	nt	*cpsE*	PM	19	19
ES-PW-033	Human	Colonization	III	nt	*cpsE*	PM	new	19
ES-PW-080	Human	Colonization	V	nt	*cpsE*	PM	2	1
ES-PW-156	Human	Colonization	III	nt	*cpsE*	PM	19	19
SH0702	Human	Colonization	V	nt	*cpsE*	PM	new	1
294206	Human	Adult ID	V	nt	*cpsG*	PM	2	1
384541	Human	Adult ID	V	nt	*cpsG*	PM	2	1
SH0334	Human	Adult ID	III	nt	*cpsG*	PM	19	19
SH0660	Human	Adult ID	V	nt	*cpsG*	PM	2	1
SH0668	Human	Adult ID	V	nt	*cpsG*	PM	223	23
BE-PW-080	Human	Colonization	III	nt	*cpsG*	PM	484	17
DK-PW-060	Human	Colonization	V	nt	*cpsG*	PM	new	19
ES-PW-153	Human	Colonization	Ia	nt	*cpsG*	PM	24	23
383728	Human	Adult ID	Ia	nt	*cpsI*	PM	23	23
290183	Human	Colonization	Ia	nt	*cpsI*	PM	23	23
GB-PW-110	Human	Colonization	Ia	nt	*cpsI*	PM	4	1

ML30419	Human	Colonization	III	nt	*cpsI*	PM	17	17
DK-PW-066	Human	Colonization	III	nt	*cpsJ*	PM	19	19
USS-107	Human	Colonization	III	nt	*cpsJ*	PM	182	19
408523	Human	Adult ID	V	nt	*cpsM*	PM	2	1
426748	Human	Adult ID	V	nt	*cpsM*	PM	24	23
SH1370	Human	Adult ID	V	nt	*cpsM*	PM	1	1
343959	Human	Adult ID	Ia	nt	*neuD*	PM	23	23
IT-PW-0063	Human	Colonization	III	nt	*neuD*	PM	17	17
446329	Human	Colonization	V	nt	promoter	PM	19	19
DK-PW-014	Human	Colonization	II	nt	promoter	PM	520	19
ES-PW-087	Human	Colonization	II	nt	promoter	PM	19	19
DK-B-USS-055	Bovine	Mastitis	Ib	nt	*cpsE, cpsH,neuD*	PM, IND, IS1381	6	6_8_10
GB-PW-013	Human	Colonization	Ib	nt	*cpsG,neuD*	PM, IS1381	8	6_8_10
418849	Human	Adult ID	II	nt	*cpsG, cpsI, cpsQ*	PM, ISSa4, ISSa4	2	1
BE-PW-078	Human	Colonization	V	nt	*cpsO,neuD*	PM, ISSpo8	530	1
BE-PW-110	Human	Colonization	V	nt	*cpsE, cpsI*	PM, PM	1	1
299693	Human	Adult ID	III	nt	*cpsG, cpsI*	PM, PM	new	17
SH2898	Human	Adult ID	V	nt	*cpsM, cpsO*	PM, PM	1	1
IT-PW-0070	Human	Colonization	Ia	nt	*cpsE, cpsH, neuC*	PM, PM, IND	7	6_8_10
GB-PW-106	Human	Colonization	Ib	nt	*cpsH, cpsJ,neuD*	PM, PM, IND	538	6_8_10
DK-B-USS-225	Bovine	Mastitis	II	nt	*cpsA, cpsB, cpsE, cpsI*	PM, PM, IND, ISSag11	489	61_67
GB-PW-087	Human	Colonization	III	nt	*cpsA, cpsD, cpsG*	PM, PM, IS1548	19	19
DK-B-USS-146	Bovine	Mastitis	II	nt	*cpsE, cpsQ, cpsJ-cpsP-cpsQ*	Unknown IS, ISSag11, DEL	n.a.	61_67
